# The Role of Microbiome and Diet on Disease Activity and Immune–Inflammatory Status in Rheumatoid Arthritis

**DOI:** 10.3390/nu18091325

**Published:** 2026-04-22

**Authors:** Aleksandra Rodziewicz, Ewa Bryl

**Affiliations:** 1 Laboratory of Photobiology and Molecular Diagnostics, University of Gdańsk, 80-309 Gdańsk, Poland; 2 Department of Physiopathology, Medical University of Gdańsk, 80-210 Gdańsk, Poland

**Keywords:** rheumatoid arthritis, diet, microbiome, vitamin D, Mediterranean diet, EPA, DHA

## Abstract

Rheumatoid arthritis (RA) is a chronic inflammatory disease of autoimmune background and unknown etiology. The importance of genetic factors in RA development is well-established. Environmental factors have also been extensively researched in relation to risk of RA and managing its symptoms. Smoking, physical activity, diet, and gut microbiota are considered to be the most essential modifiable factors in RA. Among dietary interventions, the most researched is Mediterranean diet, monounsaturated fatty acids, fish consumption, and fish oil (EPA, eicosapentaenoic acid and DHA, that is, docosahexaenoic acid). Others concerned gluten-free and vegan or vegetarian diet, salt intake, supplementation with vitamin D, antioxidants, prebiotics, and probiotics. Diet modifications can alter the gut environment, and the association between RA development or severity and the composition of gut bacteria has already been shown. This review focuses on effectiveness and usefulness of various dietary approaches and supplements in RA prevention and management, including the influence on disease activity and inflammatory status. The composition of gut microbiota and its changes in response to dietary factors are also considered. There is a great need for further research into mutual dependencies of diet, microbiome, and RA activity. The current state of knowledge provides promising evidence for future nutrition and microbial therapies.

## 1. Introduction

Rheumatoid arthritis (RA) is a chronic inflammatory autoimmune disease of unknown etiology. It is characterized by autoantibodies, synovial inflammation, and progressive joint destruction. The prevalence of disease is 0.5–1% in developed countries, and it affects women more often than men, with a female-to-male ratio of approximately 3:1 [1].

The genetic background of RA has been extensively investigated [2]. However, environmental factors play an essential role in the disease onset, progression, and response to therapy. Smoking is the best-established factor contributing to disease occurrence, worse prognosis, and response to therapy. Symptoms occurring in the course of the disease significantly reduce the quality of life. Patients suffer from pain, fatigue, sleep disturbances, and severe physical limitations. As a result, their physical activity is predominantly insufficient, which affects cardiovascular health [3]. RA is also associated with high risk of comorbidities such as hypertension, stroke, gastrointestinal disease, and osteoporosis. Patients’ sedentary lifestyle often leads to obesity, which is associated with poorer therapy effects and a higher prevalence of additional disorders [4]. Systemic inflammation associated with obesity may further aggravate RA severity and contribute to worse disease outcomes. These observations have increased interest in adjunctive lifestyle strategies, including nutritional approaches, that may help modulate not only clinical symptoms and metabolic health but also immune–inflammatory pathways involved in RA.

Apart from reducing body mass and BMI, other dietetic interventions and supplementation can help alleviate symptoms of RA. The most researched approaches include the Mediterranean diet and its elements such as monounsaturated fatty acids and fish oil (EPA, eicosapentaenoic acid, and DHA, that is, docosahexaenoic acid). Other tested interventions include gluten-free and vegan or vegetarian diet, salt intake, supplementation with vitamin D, antioxidants, prebiotics, and probiotics. These approaches are of interest not only because they may affect clinical symptoms, but also because they may interact with immune and metabolic pathways relevant to RA.

There is also growing evidence that alterations in gut microbiome composition are closely linked to the development, activity, and progression of rheumatoid arthritis. Multiple studies have demonstrated associations between specific bacterial taxa, reduced microbial diversity, and markers of systemic inflammation, autoantibody production, and joint damage. Since dietary patterns are among the strongest modulators of gut microbial composition and metabolic activity, nutritional interventions may influence RA both directly and indirectly through microbiome-related mechanisms. These mechanisms may involve the modulation of intestinal barrier integrity, cytokine production, oxidative stress, microbial metabolite signaling, and T cell polarization, all of which are relevant to the immune–inflammatory processes underlying RA.

Therefore, the aim of this review is not only to summarize the current evidence on dietary interventions in RA but also to integrate this evidence with current knowledge on gut microbiota, probiotics/prebiotics, and diet–microbiome interactions as interconnected components of RA pathogenesis and disease modulation. Particular attention is given to potential immunological mechanisms linking diet, microbial composition, and microbial metabolites with clinical outcomes, as well as to the current limitations that prevent translation of these findings into precise dietary recommendations.

## 2. Dietary Regimens and Supplements in Prophylaxis and Treatment of Rheumatoid Arthritis

Chronic inflammation in rheumatoid arthritis is sustained by dysregulation of immune pathways, including NF-κB activation, the IL-23/IL-17 axis, and NLRP3 inflammasome signaling, accompanied by increased oxidative stress and excessive production of reactive oxygen species (ROS). As emphasized in a recent comprehensive review by Kupczyk et al., redox imbalance not only amplifies cytokine production (TNF-α, IL-6, and IL-1β) but also perpetuates synovial inflammation and tissue damage [5].

Diet has emerged as a modifiable factor capable of influencing these interconnected inflammatory and oxidative pathways. According to current evidence, nutritional patterns rich in omega-3 fatty acids, fiber, polyphenols, and antioxidant vitamins may attenuate NF-κB activation, modulate Th17/Treg balance, and reduce oxidative stress markers. Importantly, many of these effects appear to be mediated indirectly through alterations in gut microbiota composition, short-chain fatty acid production, and intestinal barrier integrity, thereby linking dietary exposure to systemic immune regulation.

Although mechanistic data are increasingly consistent, clinical studies remain heterogeneous and vary in methodological quality. The main limitations include small sample sizes, short intervention duration, female-predominant cohorts, frequent use of multicomponent interventions, and limited integration of clinical outcomes with microbiome or metabolite measurements. Therefore, while dietary strategies cannot replace pharmacological therapy, they may represent a rational adjunct approach targeting immune and oxidative mechanisms involved in RA pathogenesis. The following sections summarize current evidence regarding specific dietary patterns and supplements investigated in RA prevention and treatment, with particular attention to their potential immunomodulatory and antioxidant effects.

### 2.1. Vitamin D

Vitamin D is a hormone produced in the skin from 7-dehydrocholesterol after sunlight exposure. Vitamin D is also present in food products such as fish, milk, cheese, and eggs. However, the amount in food is low, so the skin synthesis remains the main source. Biologically active form, 1,25-dihydroxyvitamin D, is known for its role in calcium metabolism and bone homeostasis. Moreover, it takes part in the regulation of the immune system. It has been shown that vitamin D modulates B and T lymphocyte function, affects antigen presentation by dendritic cells, promotes monocytic differentiation to macrophages, and regulates macrophage responses. It also indirectly affects interleukin production, e.g., inhibits the expression of the IL-6 protein, which stimulates T helper 17 (Th17) cells—crucial part of the autoimmune reaction. Therefore, proper levels of vitamin D may be essential in autoimmune diseases such as multiple sclerosis, diabetes mellitus, and rheumatoid arthritis [6].

Vitamin D status has been investigated as a potential factor associated with the risk of developing RA. A 2012 meta-analysis reported an inverse association between vitamin D intake and RA incidence, with the highest total vitamin D intake associated with a 24.2% lower risk compared with the lowest intake category. A similar relationship was observed for supplemental vitamin D intake, with the highest intake associated with a 23.6% lower risk of RA [7].

Several studies suggest that lower serum vitamin D levels are associated with higher rheumatoid arthritis activity. Two meta-analyses reported that patients with RA had lower vitamin D concentrations than healthy controls and that serum 25(OH)D was inversely associated with disease activity, particularly DAS28, and in one analysis also CRP [8,9]. However, evidence from individual observational studies remains inconsistent, as several cross-sectional studies did not confirm a significant association between vitamin D status and DAS28 or other clinical markers of disease severity [10,11,12,13,14].

Interventional evidence is also mixed. Some supplementation studies in patients with RA and vitamin D deficiency reported improvement in DAS28 after 12 weeks or 3 months of treatment [15,16], whereas a placebo-controlled trial in early RA found improvement in global health but no significant effect on DAS28, pain, or CRP [17].

**Figure 1:**
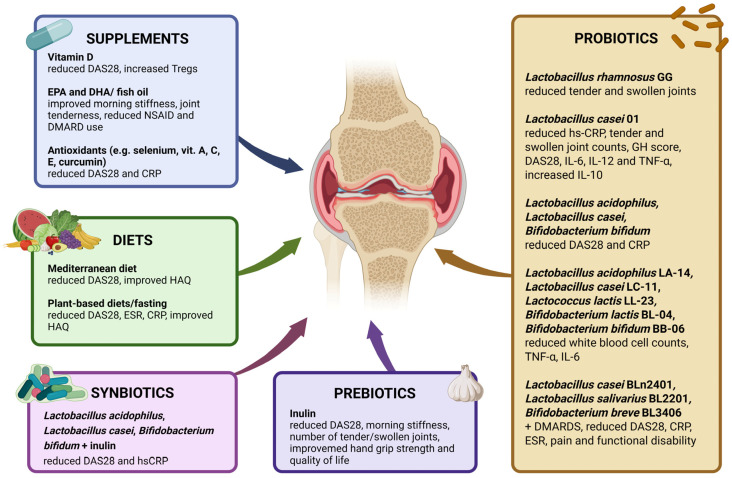
Dietary and microbiome-related interventions in rheumatoid arthritis. Summarized current evidence from selected key studies, showing the main reported effects of nutritional and microbiome-modulating strategies on disease activity, inflammatory markers, symptoms, functional status, and selected metabolic outcomes in rheumatoid arthritis. CRP, C-reactive protein; DAS28, 28-joint count disease activity score; DHA, docosahexaenoic acid; DMARDs, disease-modifying anti-rheumatic drugs; EPA, eicosapentaenoic acid; ESR, erythrocyte sedimentation rate; GH, global health; HAQ, Health Assessment Questionnaire; IL-6, interleukin 6; IL-10, interleukin 10; IL-12, interleukin 12; NSAIDs, nonsteroidal anti-inflammatory drugs; TNF-α, tumor necrosis factor α. Created in BioRender. Rodziewicz, A. (2026) https://BioRender.com/gi4xo9c (accessed on 12 April 2026).

Overall, current evidence suggests a possible inverse association between vitamin D status and RA activity; however, causality remains uncertain. Reverse causation cannot be excluded, as patients with more active disease may have reduced mobility, lower sunlight exposure, and consequently lower endogenous vitamin D synthesis. Although intervention studies are relatively few and generally small, vitamin D supplementation seems to be beneficial in RA (Figure 1). Larger well-designed placebo-controlled trials are needed to determine whether supplementation has a clinically meaningful effect on RA activity and to establish the serum vitamin D levels required for potential therapeutic benefit. The strength of evidence is low-to-moderate, as most observational data are cross-sectional and intervention trials are relatively few, small, and methodologically heterogeneous. Evidence regarding the association between serum vitamin D levels and RA activity is presented in Table 1, while the data addressing the relationship between vitamin D status and the risk of developing RA are summarized in Table 2. Findings from interventional studies evaluating the effects of vitamin D supplementation on RA activity are outlined in Table 3.

### 2.2. Mediterranean Diet

The Mediterranean diet is derived from habits and traditional foods consumed in Mediterranean countries. It is characterized by a frequent consumption of olive oil and a high proportion of plant foods such as vegetables, fruits, unrefined grains, legumes, nuts, and seeds. It also includes many condiments and spices, moderate amounts of fish, meat, eggs, and fermented dairy, as well as modest red wine intake. This dietary pattern has high fat content (up to 40–50% of total daily calories) but restricted saturated fat content (less than 8% of daily calories).

Due to its anti-inflammatory potential, Mediterranean diet is extensively researched in regard to many diseases and its prevention. This diet has been shown to have numerous beneficial health effects, especially concerning cardiovascular diseases, various types of cancer, diabetes, obesity, and metabolic syndrome [18].

Evidence from intervention studies suggests that the Mediterranean diet may improve selected clinical outcomes in RA, although results remain heterogeneous. Several randomized and controlled trials reported improvements in disease activity, pain, physical function, and quality of life following Mediterranean diet-based interventions [19,20,21]. However, many of these studies were small, of short duration, and conducted predominantly on women. In addition, some interventions combined dietary modification with exercise or physical activity promotion, which makes it difficult to isolate the effect of the diet itself [21,22].

In the MEDRA trial, both the Mediterranean diet and healthy eating guidance improved outcomes, suggesting that an improvement in overall diet quality under professional support may also contribute to clinical benefit [23].

In another randomized feeding trial on overweight and obese patients with RA, the Mediterranean diet was associated with greater reductions in DAS28 and ESR than a low-fat high-carbohydrate diet, despite similar weight loss [24]. Prospective evidence for RA prevention is less convincing. Large cohort and nested case–control studies did not show a clear association between Mediterranean diet adherence and the overall risk of developing RA [25,26,27], although one study suggested a possible protective association among ever-smokers [27].

Potential mechanisms underlying the beneficial effects of the Mediterranean diet in RA include higher intake of fiber, polyphenols, unsaturated fatty acids, and antioxidants, which may influence gut microbiota composition and modulate key inflammatory pathways involved in RA, including cytokine production (e.g., TNF-α, IL-6, and IL-1β) and T cell polarization [28,29]. Overall, the Mediterranean diet appears to be a promising complementary strategy in RA, but current evidence may be considered low-to-moderate, limited mainly by small sample sizes, short follow-up, predominantly female cohorts, and frequent co-interventions, which make it difficult to isolate the diet-specific effect. Larger well-designed trials are needed to clarify its effects on disease activity and to distinguish diet-specific effects from those of broader lifestyle change. Evidence concerning the association between adherence to the Mediterranean diet and the risk of RA development is summarized in Table 2, whereas studies evaluating its effects on disease activity are presented in Table 4.

### 2.3. Fish and Omega-3 Fatty Acids

Fatty fish are the main dietary source of eicosapentaenoic acid (EPA) and docosahexaenoic acid (DHA), two long-chain omega-3 fatty acids with well-established anti-inflammatory properties [30]. In RA, their potential immunomodulatory effects may involve the suppression of pro-inflammatory cytokine signaling, competition with arachidonic acid for eicosanoid synthesis, reduction in lipid mediators that promote synovial inflammation, and modulation of broader immunometabolic pathways.

Because habitual fish and omega-3 intake often remains below recommended levels in many developed countries, supplementation has been considered as a potential adjunctive strategy in inflammatory diseases such as RA [30]. In contrast, plant-derived α-linolenic acid (ALA) can be converted into EPA and DHA only to a very limited extent [31]. The balance between omega-3 and omega-6 fatty acids may also be relevant, as eicosanoids derived from arachidonic acid can contribute to inflammatory processes involved in RA; accordingly, one study suggested that reducing arachidonic acid intake may enhance the clinical effects of fish oil supplementation [32].

Evidence from intervention studies suggest that marine-derived omega-3 fatty acids may improve selected clinical outcomes in RA, although the magnitude and consistency of these effects vary across studies. Randomized controlled trials reported improvements in morning stiffness, joint tenderness, global disease activity, remission rates, and/or reduced NSAID or DMARD requirements following fish oil supplementation, particularly when used as adjunctive therapy [33,34,35,36]. Meta-analyses generally support a beneficial effect of omega-3 fatty acids on pain-related outcomes, morning stiffness, and NSAID use, especially when supplementation lasted at least 3 months and provided at least 2.7 g/day of EPA and DHA, although the effects on other clinical and inflammatory parameters were less consistent, and the overall quality of trials was considered low [37,38,39]. These effects may be partly explained by altered eicosanoid production and attenuation of inflammatory signaling, although not all studies demonstrated significant changes in conventional biomarkers such as CRP, IL-6, IL-1, or TNF-α [39].

Compared with marine-derived omega-3 fatty acids, evidence for plant-derived omega-3 fatty acids in RA is much more limited. In one 12-week randomized controlled trial, flaxseed supplementation, alone or combined with an anti-inflammatory diet, improved DAS28, pain, morning stiffness, and quality of life, despite no significant changes in classical inflammatory markers [40]. Beyond supplementation, observational data suggest that habitual fish consumption may also be associated with lower disease activity, as more frequent fish intake was linked to lower DAS28-CRP in one cross-sectional study [41].

In addition, two complementary analyses from the PIRA cross-over trial showed that although fatty fish meals did not acutely alter postprandial IL-6 compared with red meat or soy, they induced a distinct metabolomic profile, suggesting potential effects on immunometabolic pathways not captured by classical short-term inflammatory markers [42,43].

Evidence regarding the role of fish and omega-3 fatty acids in RA prevention remains inconsistent. A dose–response meta-analysis found a weak, non-significant inverse association between fish consumption and RA risk overall [44], while concerns have been raised that contaminants such as polychlorinated biphenyls in fatty fish may partly confound this relationship [45]. Some observational studies suggested that higher long-term intake of dietary omega-3 fatty acids or fish may be associated with a lower risk of RA [46,47], whereas a large prospective cohort did not confirm an overall protective effect of fish or omega-3 intake, although an interaction with smoking was observed in younger women [48].

Evidence on the association between EPA and DHA (fish oil) supplementation and the risk of RA development is summarized in Table 2, whereas the data from studies assessing its effects on RA disease activity are presented in Table 3.

### 2.4. Vegan Diet, Gluten-Free Diet, and Fasting

Several other dietary patterns have been investigated in rheumatoid arthritis, although the evidence remains limited because most studies were small and often involved complex or multicomponent interventions. Among these approaches, vegan and plant-based diets have been studied most extensively after the Mediterranean diet.

Early studies suggested that vegan diets may improve selected clinical outcomes in RA. A 4-week study of a very low-fat vegan diet reported reductions in joint tenderness, joint swelling, pain, and functional limitation, although no significant changes were observed in ESR, CRP, rheumatoid factor, or morning stiffness duration; importantly, the lack of a control group substantially limits interpretation of these findings [49]. More recently, the randomized controlled Plants for Joints trial showed that a multidisciplinary lifestyle intervention based on a whole-food plant-based diet, physical activity, and stress management significantly reduced DAS28 compared with usual care, while also improving body weight, fat mass, waist circumference, HbA1c, and LDL cholesterol. Notably, these clinical benefits occurred without significant between-group changes in CRP or ESR, suggesting that mechanisms beyond measurable systemic inflammation, potentially involving immunometabolic or broader lifestyle-mediated pathways, may contribute to disease improvement [50].

Raw vegan diets have also been evaluated. In one controlled trial, patients receiving a raw vegan “living food” diet reported subjective improvement in RA symptoms, which was supported by improvement in the relative activity index [51]. However, an earlier study raised concerns regarding insufficient energy availability, as patients lost substantial body weight despite increased energy intake during the intervention [52].

These findings suggest that although raw vegan diets may reduce symptoms in some patients, their nutritional adequacy and long-term feasibility remain uncertain.

Gluten-free vegan diets have likewise shown some promise, although the evidence remains mixed. In a 1-year trial, a gluten-free vegan diet was associated with a higher proportion of ACR20 responders than a well-balanced non-vegan diet, but it did not prevent radiographic progression of joint damage [53]. In another study, the same dietary approach improved DAS28, HAQ, and CRP and also favorably affected cardiovascular risk-related parameters, including reductions in total cholesterol, LDL, and BMI, together with an increase in natural atheroprotective anti-phosphorylcholine IgM antibodies [54]. These findings suggest that part of the benefit of gluten-free vegan diets may involve not only symptom reduction but also modulation of immune-related and cardiometabolic pathways. By contrast, a secondary analysis of the BELIEVE cohort found no association between habitual gluten intake and treatment response, disease activity, or CRP in patients with RA initiating biologic therapy, arguing against a meaningful short-term effect of gluten intake itself in this setting [55].

Fasting is another intensively studied dietary intervention in RA, most often combined with subsequent vegan or vegetarian refeeding [56]. Classical trials using 7–10 days of fasting followed by a gluten-free vegan diet and later a lactovegetarian diet reported substantial improvements in tender and swollen joint counts, pain, morning stiffness, grip strength, HAQ, ESR, CRP, and white blood cell count, with some benefits maintained over time [57]. Mechanistic observations from another study suggested that fasting, but not an isocaloric ketogenic diet, reduced serum IL-6 and improved ESR, CRP, and tender joint count; the authors proposed that reduced IL-6 production in the synovium might contribute to the clinical response, although the benefits appeared short-lived after fasting cessation [58].

More recent studies have also suggested that fasting-based strategies may provide rapid clinical improvement. In the NutriFast trial, a 7-day medically supervised fast followed by a plant-based diet with time-restricted eating did not outperform a guideline-based anti-inflammatory diet for the primary endpoint, but it induced rapid early improvements in functional status and disease activity, together with favorable effects on body weight and atherogenic lipoproteins [59]. Similarly, two reports from the same randomized controlled trial showed that intermittent fasting (16:8) improved DAS28, CDAI, HAQ-DI, body weight, and BMI in overweight and obese postmenopausal women with RA despite no significant between-group changes in ESR, hs-CRP, IL-6, or oxidative stress markers [60,61]. This pattern again suggests that fasting-related benefits may be mediated predominantly through weight-related, metabolic, or functional mechanisms rather than through direct short-term suppression of systemic inflammatory or oxidative pathways.

Overall, the strength of evidence for vegan, gluten-free, and fasting-based interventions remains low owing to small and often selected cohorts, complex multicomponent interventions, limited blinding, and uncertainty regarding long-term adherence and durability of effects. Their potential benefits may involve not only changes in clinical symptoms but also immunometabolic modulation, cytokine-related pathways, and cardiometabolic effects. However, many of these interventions are difficult to sustain long term, and further well-designed trials are needed before firm dietary recommendations can be made. Research on the impact of these dietary regimens on RA activity is summarized in Table 4.

### 2.5. Antioxidants

Reactive oxygen and nitrogen species generated during chronic inflammation are thought to contribute to the pathogenesis of rheumatoid arthritis by promoting oxidative stress and sustaining inflammatory responses. Accordingly, antioxidants have been investigated as potential agents for both RA prevention and symptom control, although findings from human and animal studies remain inconsistent [62,63,64].

Several studies have evaluated combined antioxidant supplementation in RA, with some promising results [65,66]. In one trial, supplementation with selenium, zinc, and vitamins A, C, and E for 12 weeks significantly reduced DAS28 and serum hs-CRP in women with RA while also increasing erythrocyte antioxidant defenses, including catalase, glutathione peroxidase, superoxide dismutase, and total antioxidant capacity [67]. These findings suggest that the modulation of oxidative stress pathways may contribute to clinical improvement, although effects on swollen and painful joint counts were not significant.

Natural antioxidant-rich foods, herbs, and spices have also been examined in relation to RA activity. In a 90-day study, cranberry juice supplementation reduced DAS28 and anti-CCP levels, although no significant changes were observed in inflammatory biomarkers and the response varied considerably between individuals [68]. This heterogeneity suggests that factors such as genetic background, environmental exposures, or gut microbiota composition may influence the clinical response to antioxidant-rich interventions. In another trial, cranberry juice combined with fish oil produced greater reductions in ESR, CRP, IL-6, DAS28-CRP, and adiponectin than fish oil alone, suggesting additional anti-inflammatory effects of this combination [69].

Curcumin, the main bioactive compound of turmeric (Curcuma longa), is one of the best-studied antioxidant compounds in RA. Its proposed mechanisms include the modulation of NF-κB signaling, regulation of pro-inflammatory cytokine production, and inhibition of phospholipase A2, COX-2, and 5-LOX activity. In a randomized clinical trial, curcumin improved DAS28 and ACR response rates with better results than diclofenac sodium and a significant reduction in CRP observed only in the curcumin group [70]. A later study using a highly bioavailable curcumin formulation also showed improvements in DAS28, VAS, and ACR scores, together with reductions in ESR, CRP, and rheumatoid factor, supporting the potential of curcumin to modulate both oxidative and inflammatory pathways relevant to RA [71].

Antioxidant-rich beverages have also been investigated in relation to RA risk. In a population-based case–control study, high tea consumption was inversely associated with RA risk among smokers and in the ACPA-positive subset of RA, suggesting that antioxidant exposure may interact with specific inflammatory or serological contexts [72].

Beyond individual compounds, the broader inflammatory or antioxidant potential of the diet has also been examined. An anti-inflammatory dietary pattern reduced DAS28-ESR in one study, although the effect was not statistically or clinically robust [73]. Similarly, the analysis of the dietary inflammatory index indicated that a more anti-inflammatory dietary profile was associated with the maintenance of low disease activity in RA [74].

Overall, available evidence suggests that antioxidant interventions may improve selected clinical and inflammatory outcomes in RA, potentially through effects on oxidative stress, cytokine signaling, and related immune pathways. However, the evidence remains limited and heterogeneous, and further well-designed studies are needed to determine which antioxidant strategies are clinically meaningful and sustainable. Interventions evaluating the use of antioxidants in RA and their effects on disease activity are summarized in Table 3, whereas the evidence on tea consumption and RA incidence is outlined in Table 2.

### 2.6. Salt

Growing evidence suggests that excessive salt intake may contribute to the development of autoimmune diseases and potentially also to disease severity. Mechanistically, high sodium chloride concentrations have been shown to promote the differentiation of T helper cells toward a pro-inflammatory Th17 phenotype through the activation of the salt-sensing serum glucocorticoid kinase 1 (SGK1) pathway [75,76].

Epidemiological evidence linking salt intake with RA risk remains inconsistent. In a Swedish nested case–control study, no significant overall association between sodium intake and RA development was observed; however, after adjustment for smoking, a twofold increase in RA risk and a dose-dependent association were found among smokers with higher sodium intake [77]. By contrast, another nested study reported a dose-dependent association between sodium intake and RA risk, with the highest risk observed in individuals consuming more than 4.55 g of sodium per day, although, in this study, the association was stronger in nonsmokers [78].

Data on salt intake and RA activity are also limited. In one study, 24 h urinary sodium excretion was significantly higher in patients with untreated early RA than in matched healthy controls and was also higher in patients with erosive disease, although no correlation with overall disease activity was found [79]. In a small dietary intervention study, low-sodium intake was associated with a reduction in the proportion of Th17 cells, whereas the restoration of normal sodium intake was followed by the opposite trend; regulatory T cells showed an inverse pattern. Although these changes were not statistically significant, the intervention was also associated with reductions in TGFβ1 and IL-9, suggesting that sodium intake may influence immune pathways relevant to RA pathogenesis [80].

Overall, current evidence suggests a possible role of high sodium intake in RA development and immune dysregulation, but available data are limited and partly contradictory. The data regarding the association between dietary salt intake and RA incidence as well as disease activity are presented in Table 2 and Table 3, respectively.

**Table 2:** Impact of dietary regimens and dietary factors on risk of RA incidence.

Factor	Study Design	Participants	Main Findings	Reference
Vitamin D	Meta-analysis	215,757 participants, 874 incident RA cases	Higher total and supplemental vitamin D intake was associated with lower RA risk.	[7]
Mediterranean diet	Prospective cohort study	174,638 women, 913 incident RA cases	No significant correlation.	[25]
	Nested case–control study	1886 controls, 368 RA cases	No significant correlation.	[26]
	Prospective cohort study	62,629 women, 480 incident RA cases	No association overall; inverse association among ever-smokers.	[27]
Fish and omega-3 consumption	Meta-analysis	174,701 participants, 3346 RA cases	No significant overall association; lower RA risk suggested with fish intake up to 2 servings/week.	[44]
	Prospective cohort study	32,232 women, 205 RA cases	Omega-3 intake > 0.21 g/day and fish intake ≥ 1 serving/week associated with lower RA risk.	[46]
	Population-based case–control study	1245 controls, 324 incident RA cases	Higher fish-derived omega-3 intake was associated with lower RA risk.	[47]
	Prospective cohort study	166,013 women, 1080 incident RA cases	No protective impact of fish or omega-3 fatty acids consumption on RA risk.	[48]
PCBs	Prospective cohort study	1721 adults	Higher PCB exposure was associated with higher RA risk in women.	[45]
Tea	Population-based case–control study	4661 controls, 2237 incident RA cases	High tea consumption was associated with lower RA risk among smokers and in ACPA-positive RA.	[72]
Salt	Nested case–control study	1886 controls, 386 RA patients	No significant association between sodium intake and RA risk.	[77]
	Cross-sectional nested study	18,555 individuals, 392 incident RA cases	Higher sodium intake was associated with higher RA risk in a dose-dependent manner.	[78]

ACPA, anti-citrullinated protein autoantibody; PCBs, polychlorinated biphenyls.

**Table 3:** Impact of dietary factors and supplements on RA activity.

Factor	Study Design	Intervention	Duration	Participants	Main Findings	Reference
Vitamin D	Exploratory study	60,000 IU/week for 6 weeks, followed by 60,000 IU/month	12 weeks	59 RA patients with vitamin D deficiency	Improvement in DAS28-CRP and DAS28-ESR.	[15]
	Randomized placebo- controlled trial	300,000 IU (a single dose)	3 months	39 early RA patients	Improvement in global health but no significant effect on DAS28, pain, or CRP.	[17]
	Randomized, interventional study	50,000 IU of vitamin D2 weekly	3 months	40 RA patients	Reduction in DAS28 and increase in Tregs.	[16]
*n*-3 fatty acids + low AA diet	Randomized double-blind cross-over study	Low-AA anti-inflammatory diet + *n*-3 fatty acids (30 mg per kg of body weight/day)	8 months	60 RA patients	Greater reduction in tender and swollen joints with low-AA diet plus *n*-3 fatty acids.	[32]
EPA and DHA/fish oil supplementation	Randomized, double-blind trial	2 g EPA + 1.2 g DHA/day	12 weeks	51 RA patients	Improved morning stiffness, joint tenderness, and CRP.	[33]
	Double-blind, controlled study	10 g fish oil/day	6 months	43 RA patients	Reduced NSAID use; improved global arthritic activity.	[34]
	Double-blind placebo controlled study	1.71 g EPA + 1.14 g DHA/day	15 months	52 mild RA patients	Reduced NSAID requirement.	[35]
	Randomized, double-blind controlled trial	5.5 g EPA and DHA/day	12 months	121 patients with recent onset RA	Higher remission rate and lower DMARD use.	[36]
	Meta-analysis	≥2.7 g/day EPA + DHA	3–4 months	823 patients with inflammatory joint pain (including RA)	Improved patient-reported pain, morning stiffness, tender joints, and NSAID use.	[37]
	Meta-analysis	≥2.7 g/day omega-3 fatty acids	≥3 months	370 RA patients	Reduced NSAID dose.	[38]
	Meta-analysis	0.30–9.60 g/day omega-3 fatty acids	12–72 weeks	1252 RA patients	Improved morning stiffness, tender joint count, ESR, and pain; reduced leukotriene B4.	[39]
Flaxseed + anti- inflammatory diet	Randomized controlled trial	Flaxseed (30 g/day) with or without anti-inflammatory diet	12 weeks	120 RA patients	Improved DAS28, pain, morning stiffness and quality of life; no significant change in ESR or CRP.	[40]
Red meat/fatty fish/soy protein-based meals	Randomized cross-over trial	Isocaloric meals based on red meat, fatty fish, or soy protein	Acute postprandial study	25 RA patients	No significant differences in postprandial IL-6 between meals.	[42,43]
Fish oil + cranberry juice	Randomized, single-blind intervention study	3 g/day omega-3 fatty acids alone or with 500 mL/day cranberry juice	90 days	62 RA patients	Fish oil plus cranberry juice reduced ESR, CRP, IL-6, DAS28-CRP, and adiponectin; fish oil alone reduced DAS28-CRP and adiponectin.	[69]
Antioxidants	Double-blind, placebo-controlled trial	200 µg selenium-enriched yeast vs. placebo	90 days	55 patients with moderate RA	No significant differences between groups.	[62]
	Randomized controlled study	Standard treatment alone vs. standard treatment plus antioxidant combination vs. standard treatment plus high-dose vitamin E	2 months	30 RA patients	Improved RAI, morning stiffness, and ESR in antioxidant-supplemented groups.	[65]
	Open pilot study	Antioxidant-enriched spread plus vitamin C	10 weeks + 4-week washout	8 women with RF-positive RA	Reduced swollen and painful joints; improved general health and DAS; partial loss of benefit after washout.	[66]
	Pre-post clinical trial	Capsules containing selenium, zinc, and vitamins A, C, and E	12 weeks	39 RA patients	Reduced DAS28 and hs-CRP; increased CAT, GPx, SOD, and total antioxidant capacity; no change in swollen or painful joints.	[67]
	Randomized controlled study	500 mL/day low-calorie cranberry juice	90 days	38 women with RA	Reduced DAS28 and anti-CCP.	[68]
	Randomized, single-blind clinical study	500 mg curcumin, 50 mg diclofenac sodium, or both	8 weeks	38 patients with active RA	All groups improved DAS28 and ACR response; CRP decreased only in the curcumin group; curcumin was superior to diclofenac alone.	[70]
	Randomized double-blind placebo-controlled trial	Placebo or curcumin product 250 or 500 mg twice daily	90 days	36 RA patients	Improved DAS28, VAS and ACR scores; reduced ESR, CRP, and RF.	[71]
Salt	Pre-post clinical trial	Low-sodium diet for 3 weeks, followed by normal-sodium diet for 2 weeks	5 weeks	14 RA patients	Low sodium intake was associated with a decrease in Th17 cells and an opposite trend in Treg cells; reduced TGFβ1 and IL-9.	[80]

AA, arachidonic acid; ACR, American College of Rheumatology response criteria; CAT, catalase; CCP, cyclic citrullinated peptide; CRP, C-reactive protein; DAS28, 28-joint count disease activity score; DHA, docosahexaenoic acid; DMARDs, disease-modifying anti-rheumatic drugs; EPA, eicosapentaenoic acid; ESR, erythrocyte sedimentation rate; GPx, glutathione peroxidase; hs-CRP, high-sensitivity C-reactive protein; TGFβ1, transforming growth factor β1; IL-6, interleukin 6; IL-9, interleukin 9; NSAIDs, nonsteroidal anti-inflammatory drugs; RAI, Ritchie’s articular index; RF, rheumatoid factor; SOD, superoxide dismutase; Th17, T helper 17; Tregs, regulatory T cells; VAS, visual analog scale.

**Table 4:** Impact of dietary regimens on RA activity.

Diet	Intervention	Duration	Participants	Main Findings	Reference
Mediterranean diet	Cretan Mediterranean diet vs. regular Swedish diet	12 weeks	51 RA patients	Improved DAS28, HAQ, and SF-36; no effect on NSAID use.	[19]
	Mediterranean diet education plus cooking classes vs. general healthy eating advice	6 months	130 female RA patients	Improved pain and HAQ at 3 months; improved patient global assessment, pain, and EMS at 6 months.	[20]
	Personalized Mediterranean diet plus physical activity counseling vs. generic diet and physical activity advice	12 weeks	40 women with RA in remission	Lower DAS28; improved body composition, glucose, and vitamin D status.	[21]
	Mediterranean diet vs. low-fat high-carbohydrate diet vs. regular diet	12 weeks	154 overweight and obese RA patients	Greater reductions in DAS28 and ESR with Mediterranean diet; VAS improved vs. control.	[24]
Vegan diet	Very low-fat vegan diet; no control group	4 weeks	24 RA patients	Improved joint tenderness/swelling, pain, functional limitation, and morning stiffness severity; no significant changes in ESR, CRP, RF, or morning stiffness duration.	[49]
	Whole-food plant-based lifestyle program including physical activity and stress management vs. usual care	16 weeks	83 RA patients	Greater reduction in DAS28 vs. usual care; patient-reported outcomes favored intervention but were not statistically significant.	[50]
	Raw vegan (“living food”) diet vs. omnivorous diet	3 months	40 RA patients	Subjective symptom improvement and reduction in RAI^1^.	[51]
Vegan gluten-free diet	Gluten-free vegan diet vs. well-balanced non-vegan diet	9–12 months	47 RA patients	Higher clinical response in the vegan group; no significant difference in radiographic progression.	[53]
	Gluten-free vegan diet preceded by 1-day low-energy fast vs. well-balanced non-vegan diet	3–12 months	58 patients with active RA	Lower DAS28 and HAQ at 3 and 12 months; lower CRP at 12 months.	[54]
Gluten intake	High vs. low-to-medium habitual gluten intake	14–16 weeks	37 RA patients	No significant differences between groups.	[55]
Vegan diet and fasting	7–10 days of fasting followed by gluten-free vegan diet and then lactovegetarian diet vs. mixed diet	13 months	34 RA patients	Improved clinical indices, ESR, CRP, and WBC after 4 weeks; effects maintained throughout the year.	[57]
	7-day fast vs. isocaloric ketogenic diet; both followed by lactovegetarian refeeding	21 days	23 RA patients with active disease	Fasting, but not ketogenic diet, reduced IL-6 and improved ESR, CRP, and tender joint count.	[58]
	7-day fast followed by plant-based diet with time-restricted eating vs. guideline-based anti-inflammatory diet	12 weeks	53 RA patients	No significant between-group difference in the primary endpoint; faster early improvement in HAQ-DI and disease activity in the fasting group; both diets improved RA activity.	[59]
Intermittent fasting	16:8 intermittent fasting vs. usual diet plus healthy eating advice	8 weeks	44 overweight and obese postmenopausal women with RA	Improved DAS28, CDAI, and HAQ-DI; no significant changes in inflammatory markers.	[60,61]
Anti- inflammatory diet in RA (ADIRA)	Anti-inflammatory diet vs. control diet reflecting habitual Swedish intake	10 weeks, 4-month washout	47 RA patients	No significant difference in DAS28-ESR between diet periods; lower DAS28-ESR after intervention vs. baseline.	[73]

CDAI, Clinical Disease Activity Index; CRP, C-reactive protein; DAS28, 28-joint count disease activity score; EMS, early morning stiffness; ESR, erythrocyte sedimentation rate; HAQ, Health Assessment Questionnaire; HAQ-DI, Health Assessment Questionnaire Disability Index; IL-6, interleukin 6; NSAIDs, nonsteroidal anti-inflammatory drugs; SF-36, short form-36 health survey; RA, rheumatoid arthritis; RAI, Ritchie articular index; RAI^1^, relative activity index; VAS, visual analog scale; WBCs, white blood cells.

## 3. Gut Microbiome in Rheumatoid Arthritis

Increasing attention has been given to the gut microbiome as a potential link between environmental exposures, including diet, and immune dysregulation in rheumatoid arthritis (RA). Because dietary patterns are among the major determinants of gut microbial composition and metabolic activity, microbiome-related mechanisms may partly explain why some nutritional interventions influence RA activity, even when direct immunological effects are not fully characterized. The gut microbiota plays a central role in maintaining immune homeostasis; therefore, disturbances in its composition or function may contribute to aberrant immune responses relevant to RA pathogenesis.

In addition to taxonomic dysbiosis, intestinal barrier dysfunction has emerged as another mechanism of interest in RA. Increased gut permeability may expose the immune system to luminal microorganisms and microbial products that would not normally cross the epithelial barrier. According to the gut–joint axis hypothesis, this may promote systemic immune activation and, via hematogenous spread, contribute to local inflammatory processes within the joints [81].

Diet may also influence these processes through effects on intestinal barrier integrity. Compounds characteristic of the Western diet, including milk fat, high-fat dietary patterns, alcohol, gliadin, and selected food additives such as salt, sugar, emulsifiers, surfactants, organic solvents, and microbial transglutaminase, have been associated with the deterioration of the gut barrier structure. In contrast, several nutrients and compounds abundant in Mediterranean-style dietary patterns, including curcumin, polyphenols—such as quercetin, myricetin, and kaempferol—zinc, vitamin D, glutamine, and tryptophan, have been reported to support barrier function [82].

Patients with rheumatoid arthritis display diminished gut microbial diversity and significantly different composition of gut and oral microbiome than healthy controls. Scientists observed relative enrichment of Gram-positive bacteria and depletion of Gram-negative bacteria such as some *Proteobacteria* and Gram-negative *Firmicutes* of the *Veillonellaceae* family in the gut microbiome of RA patients. Particularly, the *Haemophilus* species were reduced and *Lactobacillus salivarius* was more abundant in RA patients. Modifications in gut microbiome make-up correlated with clinical measures such as CRP, anti-CCP, and RF. Therefore, it was suggested that chronic inflammation characteristic of rheumatoid arthritis might be exacerbated by the deficiency of commensal bacteria or overabundance of pathogenic bacteria. The oral and gut dysbiosis was partially corrected after treatment with disease-modifying anti-rheumatic drugs (DMARDs) [83].

Other studies have similarly suggested that early RA is characterized by the depletion of bacterial groups potentially involved in immune homeostasis. Vaahtovuo et al. demonstrated that patients with early RA have significantly less bifidobacteria and bacteria of the *Bacteroides-Porphyromonas-Prevotella* group, the *B. fragilis* subgroup, and the *E. rectale*—*C. coccoides* group in comparison with fecal microbiota of patients with fibromyalgia (FM). Authors emphasize that there are two possible explanations for these results. Presumably, patients with RA lack certain bacteria involved in immune homeostasis. However, it is also possible that those patients have greater diversity of bacteria that cannot be detected by the chosen method and set of used oligonucleotide probes. Authors chose FM patients as controls because, just as RA patients, they receive NSAID medication, and their age and sex distributions are similar, but FM is a noninflammatory disease [84].

Experimental and clinical data also point to enrichment of taxa with potential pro-inflammatory properties. In another experimental study, diminished gut microbial diversity in RA patients correlated with disease duration and autoantibody levels. The diversity between microbiota of RA patients and healthy controls was displayed through differences in taxa, particularly the increase in rare bacterial lineages such as phylum *Actinobacteria*, along with its two genera, *Eggerthella* and *Actinomyces*, in RA patients. Moreover, as it was shown in a humanized mouse model, *Collinsella aerofaciens* intensifies the severity of arthritis. *Collinsella* also increases gut permeability, lowers the expression of tight junctions (TJ) proteins, and stimulates the epithelial production of IL-17A, so it may cause an increased inflammation and participate in RA pathogenesis [85].

In an observational study including 110 patients with established rheumatoid arthritis, an increased abundance of the genus *Collinsella* was associated with cumulative inflammatory burden in RA. Higher levels of *Collinsella* were observed in patients with moderate-to-high disease activity and remained independently associated with long-term inflammatory activity alongside age, obesity, and impaired physical function. The same study also identified additional taxonomic shifts, including higher abundance of the minority phyla, *Spirochaetes* and *Synergistetes*, in RA, while *Oxalobacteraceae* was enriched in controls, being consistent with previous data suggesting a potentially protective association with RA. Alterations in gut microbiota composition were accompanied by differences in predicted microbial metabolic pathways, particularly those related to purine metabolism and short-chain fatty acid fermentation, alongside broader changes in biosynthetic and energy generation pathways [86].

Li et al. also demonstrated significant alterations of gut microbiota in RA patients compared to healthy controls. Phylum *Proteobacteria*, which includes many pathogenic bacteria, such as *Enterobacter*, was more abundant in RA patients, whereas *Firmicutes* was diminished. Moreover, the relative abundance of *Blautia*, *Ruminococcus2*, and *Odoribacter* was negatively correlated with T, B, CD4+T, and Tregs [87].

Beyond taxonomic composition, evidence also suggests that translocation of bacterial products may contribute to inflammatory burden in RA. In a cross-sectional clinical study including 87 patients with moderate-to-severe rheumatoid arthritis, Kitamura et al. examined associations between intestinal bacterial load, bacterial-derived substances, and disease activity. Serum levels of lipopolysaccharide-binding protein (LBP) and fecal lipopolysaccharide (LPS) were positively correlated with multiple markers of disease activity and systemic inflammation, including DAS28, CRP, ESR, MMP-3, and IL-6, while total intestinal bacterial counts were inversely associated with circulating LPS levels. In addition, antibodies against *Porphyromonas gingivalis*-derived LPS were inversely correlated with pain and patient-reported disease activity, supporting the concept of an oral–gut microbiome axis in RA. Although the study design does not allow causal inference, the findings suggest that translocation of bacterial components and impaired endotoxin neutralization may contribute to inflammatory burden in established RA [88].

The gut microbiome may also be relevant to therapeutic response. In a cross-sectional study including 94 patients with established rheumatoid arthritis treated with disease-modifying anti-rheumatic drugs (DMARDs) and 30 healthy controls, Koh et al. demonstrated that gut microbiome composition in RA differs from that of healthy individuals, despite comparable overall microbial richness and diversity. Microbiome profiles were influenced by age, with younger RA patients (<45 years) showing reduced diversity and a distinct microbial signature compared with older patients and controls, whereas disease activity, RF, and ACPA status were not associated with microbial composition. Most conventional synthetic DMARDs (csDMARDs; e.g., methotrexate, sulfasalazine) and biologic DMARDs (bDMARDs; targeted monoclonal antibodies or fusion proteins directed against specific immune pathways) showed limited impact on gut microbiota structure, although sulfasalazine was associated with reduced diversity and specific taxonomic shifts. Importantly, the relative abundance of the selected genera, particularly *Subdoligranulum* and *Fusicatenibacter*, was associated with subsequent response to second-line csDMARD therapy, suggesting a potential link between gut microbiome composition and treatment outcomes in established RA [89].

In a multicenter, longitudinal observational study of 144 DMARD-naive patients with newly diagnosed rheumatoid arthritis, Danckert et al. investigated whether baseline gut and oral microbiota could predict clinical response to DMARD therapy. Shotgun metagenomic sequencing revealed that patients who achieved a minimal clinically important improvement showed a progressive reduction in specific taxa, particularly *Prevotella* spp. at 6 weeks and *Streptococcus* spp. at 12 weeks of treatment. Importantly, baseline microbiome composition was indicative of future treatment response, suggesting that microbiota signatures precede and potentially modulate therapeutic outcomes. The authors noted that DMARD therapy was associated with a partial restoration of a more eubiotic microbiome profile, although causal relationships cannot be inferred from the observational design [90]. Together, these findings suggest that gut microbiome composition may not only reflect disease status but also influence therapeutic trajectories in RA.

In another large cross-sectional study including 262 patients with rheumatoid arthritis and 475 healthy controls, Wang et al. demonstrated that gut microbiota profiles could be used to construct predictive models discriminating RA from healthy individuals. Using 16S rRNA sequencing and machine learning approaches, the authors identified a set of seven genera that enabled moderate diagnostic performance across independent datasets. Although the study focused primarily on disease classification rather than therapeutic outcomes, several of the identified taxa (including *Subdoligranulum* and *Fusicatenibacter*) have previously been implicated in treatment response and inflammatory regulation in RA. The authors emphasized that microbiome-based prediction remains exploratory and requires validation in longitudinal and treatment-focused cohorts [91].

Growing evidence indicates that in rheumatoid arthritis, microbiome alterations are not limited to compositional dysbiosis but also involve disturbed microbial metabolic activity with direct relevance to immune regulation and epithelial barrier function [92].

In particular, short-chain fatty acids (SCFAs), which are generated by bacterial fermentation of dietary fiber, appear to represent one of the key metabolic links between diet and immune homeostasis. In patients with RA, levels of acetate, propionate, butyrate, and valerate were reduced, and acetate, propionate, and butyrate correlated positively with peripheral regulatory B cell frequencies, whereas the administration of the first three SCFAs ameliorated collagen-induced arthritis in mice and promoted Breg differentiation in an FFA2-dependent manner [93].

In a complementary study, RA patients and arthritic mice also exhibited reduced microbial-derived SCFAs, while butyrate supplementation suppressed arthritis by increasing the serotonin-derived metabolite 5-hydroxyindole-3-acetic acid (5-HIAA), thereby enhancing aryl-hydrocarbon receptor (AhR)-dependent regulatory B cell function and limiting germinal-center B cell and plasmablast differentiation [94].

Consistent with the concept that dietary substrates may influence RA through microbial metabolite production, a 28-day high-fiber dietary intervention in RA patients increased circulating Tregs, improved the Th1/Th17 balance, reduced markers of bone erosion, and improved patient-related outcomes [95].

In addition to SCFAs, altered bile acid metabolism may also contribute to microbiome-mediated effects in arthritis, as remodeling of the gut microbiota in adjuvant-induced arthritis rats was accompanied by changes in bile acid metabolism, including treatment-responsive alterations in glycochenodeoxycholic acid and glycocholic acid, together with reduced TNF-α and IL-6 levels [96].

Moreover, in a multi-omics study of untreated patients with RA, gut dysbiosis was characterized by the enrichment of *Klebsiella*, *Escherichia*, *Eisenbergiella*, and *Flavobacterium* and the depletion of *Fusicatenibacter*, *Megamonas*, and *Enterococcus*. Fecal metabolomic profiling in the same study demonstrated disturbances in tryptophan, glycerophospholipid, and unsaturated fatty acid metabolism, with reduced levels of downstream tryptophan metabolites, including 5-hydroxyindoleacetic acid (5-HIAA, a serotonin-derived tryptophan metabolite), kynurenic acid, xanthurenic acid, and 3-hydroxyanthranilic acid. Together, these findings suggest that functional metabolic alterations of the gut microbiome may contribute to immune activation in RA alongside taxonomic dysbiosis [97]. Gut microbial metabolism can also shape the biological activity of plant-derived dietary compounds and their downstream metabolite output. In an in vitro digestion–fermentation model, polysaccharides from etiolated green tea remained largely resistant to upper gastrointestinal digestion and were subsequently utilized by human fecal microbiota, leading to increased short-chain fatty acid production, lower pH, the enrichment of *Bacteroidota*, *Faecalibacterium* and *Bacteroides*, and a reduction in *Escherichia_Shigella* and *Dorea* [98]. Similarly, the digestion products of the Changyanning formula were shown to modulate fecal microbiota composition and metabolite profiles during in vitro fermentation, supporting the concept that gut microbial biotransformation may substantially influence the functional output of complex plant-derived interventions [99].

**Figure 2:**
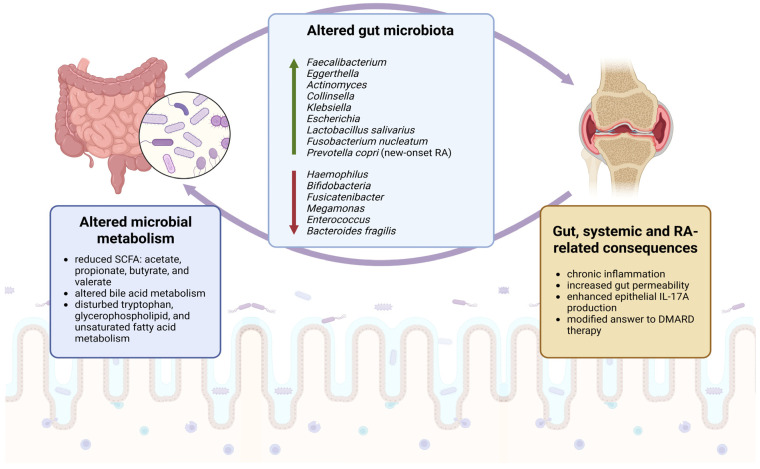
Gut microbiome dysbiosis and metabolic alterations in rheumatoid arthritis. Overview of selected alterations in gut microbial composition and metabolism reported in rheumatoid arthritis, including representative taxonomic shifts, disturbed microbial metabolic activity and metabolite profiles, and their potential contribution to intestinal barrier dysfunction, systemic inflammation, immune dysregulation, and RA-related inflammatory processes. Upward arrow indicate increased abundance, whereas downward arrow indicate decreased abundance of the indicated bacterial taxa. DMARDs, disease-modifying anti-rheumatic drugs; IL-17A, interleukin 17A; SCFA, short-chain fatty acids. Created in BioRender. Rodziewicz, A. (2026) https://BioRender.com/c34xsk4 (accessed on 12 April 2026).

Together, these findings indicate that RA-associated dysbiosis should be interpreted not only in terms of taxonomic shifts but also in relation to altered microbial metabolic activity and metabolite profiles, which may directly contribute to barrier dysfunction, immune dysregulation, and inflammatory processes in RA (Figure 2). Among the microorganisms most frequently implicated in RA pathogenesis, several have attracted particular interest because of their potential mechanistic links to Th17 responses, loss of tolerance, epithelial dysfunction, and arthritis severity.

### 3.1. Segmented Filamentous Bacteria

Segmented filamentous bacteria (SFB) are spore-forming Gram-positive commensal bacteria most closely related to the genus *Clostridium*, which colonize the ileum of the small intestine of numerous species, including humans [100]. It was shown that they are capable of inducing CD4+ T helper cells, which results in the upregulation of IL-17+Th17 cells in mice. Thus colonization with SFB may trigger inflammatory processes and contribute to the pathogenesis of autoimmune diseases [101]. This mechanism was confirmed in the K/BxN mouse model of autoimmune arthritis, where the emergence of SFB was enough to activate arthritis [102,103].

### 3.2. Prevotella

*Prevotella* species are anaerobic Gram-negative bacteria considered commensal due to their abundant colonization of healthy human body and their infrequent involvement in infection. However, increased prevalence of *Prevotella* species has been identified in many chronic inflammatory diseases, such as bacterial vaginosis, metabolic disorders, low-grade systemic inflammation, and rheumatoid arthritis [104].

*Prevotella copri* has been shown to be increased in stool samples from patients with new onset RA (NORA) in comparison with patients with chronic, treated RA (CRA), psoriatic arthritis, or healthy controls. The overgrowth of *P. copri* correlated with a reduction in *Bacteroides* and in beneficial microbes in the NORA patients [105]. Scher et al. indicate that lower prevalence of *Prevotella* in CRA than NORA may be caused by decreased inflammation and disease activity. DAS28 was slightly lower in CRA patients but the difference in CRP levels was more significant. Thus, the authors speculate on microbial modulating properties of CRP itself and emphasize the importance of future research on this subject.

Alpizar-Rodriguez et al. compared microbiome composition in 50 first-degree relatives (FDRs) of patients with RA (FDR controls) and 83 patients in pre-clinical RA stages. Significant differences were found, especially the enrichment of *Prevotella* spp., in individuals at risk for RA compared with controls [106].

Immune relevance of *Prevotella copri* in patients with RA has been proved recently. A total of 32% of RA patients were found to have serum IgA or IgG antibodies specific for *P. copri*, whereas in healthy controls and patients with other arthritic diseases, these specific antibodies were almost absent. Moreover, antibodies to the gut commensals *Bacteroides fragilis* and *Escherichia coli* were predominantly absent in all patients and healthy controls. The study demonstrates that the immune responses to *P. copri* may develop in RA patients and may be involved in disease pathogenesis or progression in some patients [107].

However, Marietta et al. showed that *Prevotella histicola* may have protective influence. HLA-DQ8 mice treated with *P. histicola* displayed significantly reduced incidence and severity of arthritis in comparison with the controls. As a consequence of treatment with *P. histicola*, an increased expression of enzymes required to produce antimicrobial peptides and of tight junctions proteins (zonula occludens 1 and occludin) was observed, which resulted in lower gut permeability. Authors ascribed immunomodulating properties to this species and indicated that it can be further explored as therapy for RA [108]. *Prevotella* consists of over 40 different species with high genetic diversity, which presumably explains this conflicting data concerning RA.

### 3.3. Subdoligranulum

Recent evidence suggests a context-dependent role of *Subdoligranulum* in rheumatoid arthritis, spanning both potentially arthritogenic and immunoregulatory mechanisms. Using clonal IgA and IgG autoantibodies derived from individuals at risk for RA, one study identified a specific strain of *Subdoligranulum* that was selectively targeted by autoreactive antibodies and promoted inflammatory arthritis in gnotobiotic mouse models, supporting a possible role in early immune priming and loss of tolerance [109].

In contrast, a recent multi-omics Mendelian randomization study combined with in vitro validation demonstrated that *Subdoligranulum* variabile exerted anti-inflammatory effects by inducing tumor necrosis factor-stimulated gene 6 (TSG-6) expression in joint cells, leading to a reduced TNF-α production and attenuation of inflammatory responses [110].

Together, these findings highlight marked strain-level heterogeneity within the genus *Subdoligranulum* and suggest that its role in RA may depend on microbial functional properties, host immune context, and disease stage rather than taxonomic presence alone.

### 3.4. Fusobacterium Nucleatum

Experimental evidence implicates *Fusobacterium nucleatum* as a potential pro-inflammatory contributor to rheumatoid arthritis pathogenesis. In a mechanistic study combining human samples and murine models, *F. nucleatum-derived* outer membrane vesicles containing the adhesin FadA were shown to aggravate inflammatory arthritis by promoting macrophage activation and increasing the production of pro-inflammatory cytokines, including TNF-α and IL-6. FadA-mediated signaling enhanced intestinal permeability and facilitated systemic dissemination of inflammatory stimuli, thereby amplifying joint inflammation. Although primarily based on experimental models, these findings support a role for specific bacterial virulence factors rather than overall bacterial abundance, in linking gut dysbiosis to immune activation in RA [111].

## 4. Microbiome-Modulating Interventions in Rheumatoid Arthritis

### 4.1. Probiotics

Probiotics are viable microorganisms with great health potential and various beneficial effects on gut microbial balance, gut permeability, immune system, and other human body function. Their efficacy has been assessed especially in inflammatory diseases and gastrointestinal disorders. Impact of probiotic supplementation has also been studied in RA. *Lactobacillus* supplementation was the most extensively examined. In one study, patients with RA received *Lactobacillus rhamnosus* GG or placebo for 12 months. There were no significant differences in the activity of RA evaluated by clinical examination, HAQ index, and laboratory tests such as ESR, CRP, and pro- and anti-inflammatory cytokines. However, more patients in the *Lactobacillus* group felt subjectively better. Also, the reduction in the mean number of tender and swollen joints was greater in the *Lactobacillus* group in comparison to the placebo group [112].

Another study evaluated influence of *Lactobacillus casei* 01 supplementation on women with RA. For 8 weeks, 22 patients in the first group received 10^8^ colony forming units (CFU) of *L. casei* 01 daily, and 24 patients in another group received placebo. Probiotic supplementation resulted in a decrease in serum high-sensitivity C-reactive protein (hs-CRP) levels, tender and swollen joint counts, global health (GH) score, and DAS28. Moreover, the level of regulatory cytokine IL-10 was increased due to *L. casei* 01 supplementation, whereas the levels of pro-inflammatory IL-12 and TNF-α were decreased [113].

Vaghef-Mehrabany E. et al. also assessed the effects of *Lactobacillus casei* 01 supplementation on RA patients and received similar results. After 8 weeks of receiving probiotic capsules, the patients exhibited a significant decrease in DAS28 and serum pro-inflammatory cytokines (IL-6, IL-12, and TNF-α), whereas IL-10 level raised when compared to the placebo group [114].

Zamani et al. in their randomized, double-blind, placebo-controlled trial used capsules with a mixture of three probiotic strains: *Lactobacillus acidophilus*, *Lactobacillus casei*, and *Bifidobacterium bifidum*. A total of 30 patients received probiotic capsules and the other 30 received placebo for 8 weeks. After the intervention, the probiotic group displayed improved DAS-28, decreased serum insulin levels, and serum hs-CRP concentrations in comparison to the placebo group [115].

Three months of supplementation with *Lactobacillus rhamnosus* GR-1 and *Lactobacillus reuteri* RC-14 did not result in clinical improvement of RA, as measured by the ACR20. Moreover, changes in cytokine levels were more beneficial in the placebo group [116].

A mixture containing five strains *Lactobacillus acidophilus* LA-14, *Lactobacillus casei* LC-11, *Lactococcus lactis* LL-23, *Bifidobacterium lactis* BL-04, and *Bifidobacterium bifidum* BB-06 (10^9^ CFU/g of each) was taken for 60 days by RA patients in the Cannarella et al. research. After 60 days, white blood cell counts, TNF-α, and IL-6 plasma levels were significantly reduced in the probiotic group. Also, the oxidative/nitrosative profile was improved—nitric oxide metabolites were lower, whereas the sulfhydryl group and total radical trapping antioxidant parameters were higher [117].

More recently, the potential role of probiotics as an adjunct to pharmacological treatment has been evaluated in patients with early rheumatoid arthritis. In a randomized, placebo-controlled trial including 100 patients with newly diagnosed RA, adjunctive supplementation with a multi-strain probiotic preparation (*Lactobacillus casei* BLn2401, *Lactobacillus salivarius* BL2201 and *Bifidobacterium breve* BL3406) in combination with conventional synthetic DMARDs resulted in faster and more sustained reductions in disease activity (DAS28), CRP, ESR, pain, and functional disability over a 12-month follow-up compared with DMARD therapy alone. Probiotic supplementation was identified as an independent predictor of achieving remission or low disease activity, and it was additionally associated with an improved quality of life and reduced corticosteroid use. However, as gut microbiota composition was not directly assessed, the mechanistic link between probiotic intake and immunomodulation in RA remains to be elucidated [118].

Overall, the evidence for using probiotics in RA treatment is promising but remains of low strength, as most studies are relatively small and do not directly confirm that clinical improvement is accompanied by measurable changes in gut microbiome composition or function.

### 4.2. Prebiotics and Synbiotics

Beyond probiotics, increasing attention has been given to prebiotics and synbiotics as strategies aimed at modulating gut microbiota function rather than introducing exogenous bacterial strains. In a randomized, double-blind, placebo-controlled trial including 60 patients with rheumatoid arthritis, supplementation with a synbiotic preparation containing *Lactobacillus acidophilus*, *Lactobacillus casei*, *Bifidobacterium bifidum*, and inulin for 8 weeks resulted in significant improvements in DAS28, serum hs-CRP, insulin resistance indices, and lipid profile compared with placebo [119].

Similarly, in a randomized controlled trial of 50 patients with RA, daily supplementation with inulin for 8 weeks led to significant reductions in DAS28, morning stiffness, number of tender/swollen joints, alongside improvements in hand grip strength and quality of life. Notably, both interventions exerted clinical and biochemical benefits despite the absence of direct microbiome sequencing, suggesting that the enhancement of microbial metabolic activity—potentially via increased short-chain fatty acid production and improved gut barrier function—may contribute to immune modulation in RA. However, the relatively short intervention periods and limited mechanistic data warrant cautious interpretation of these findings and highlight the need for integrative studies combining clinical outcomes with microbiome and metabolomic analyses [120].

Thus, the current evidence for prebiotics and synbiotics remains preliminary and is limited by short intervention periods, small sample sizes, and the lack of direct microbiome sequencing or metabolomic validation.

### 4.3. Dietary Interventions

Diet is one of the major determinants of gut microbial composition and metabolic activity and may therefore influence immune pathways relevant to rheumatoid arthritis. However, despite growing interest in nutritional strategies in RA, only a limited number of studies have simultaneously evaluated dietary intervention, gut microbiota changes, and clinical outcomes in this disease. As a result, the microbiome-mediated effects of dietary interventions in RA remain insufficiently characterized, and much of the available evidence is still indirect. This represents an important research gap, because dietary modulation of the gut microbiome may help explain at least part of the variability in clinical response to nutritional interventions.

Among the dietary patterns investigated in RA, the Mediterranean diet is one of the most promising from a clinical perspective, but its microbiome-related effects in RA have rarely been examined directly. In healthy individuals, higher adherence to the Mediterranean diet has been associated with a more favorable microbial profile, including lower *Escherichia coli* levels and a higher *Bifidobacteria*:*E. coli* ratio [121,122]. These findings support the biological plausibility of microbiome-mediated benefits, but dedicated RA studies integrating dietary intervention, microbiome analysis, and clinical outcomes are still lacking.

Some of the few direct data in RA come from plant-based dietary interventions. In patients with active RA, a shift from an omnivorous diet to a regimen consisting of fasting, followed by a vegan diet and later a lactovegetarian diet, was associated with significant alterations in intestinal bacterial profiles, and these changes were linked to clinical improvement [123]. Similarly, Peltonen et al. reported significant microbiota modifications after 1 month of a raw vegan diet in RA patients, which corresponded with improvement in disease activity [124]. Although these findings suggest that plant-based diets may modulate RA through microbiome-related mechanisms, the number of available studies remains very small, the interventions are complex, and causality cannot be established.

Gluten-free diets are sometimes discussed in the context of RA, but RA-specific data on their effects on the gut microbiome are currently lacking. In healthy subjects, short-term gluten-free diet has been associated with reductions in several bacterial groups considered beneficial, including *Bifidobacterium* and *Faecalibacterium prausnitzii* [125,126]. Therefore, the microbiome-related relevance of gluten restriction in RA remains uncertain.

Recent evidence also suggests that not only dietary composition but also meal timing may influence gut microbiota–host interactions in rheumatoid arthritis. In an interventional study, time-restricted feeding modulated diurnal oscillations of gut microbiota and microbial metabolites, which were aligned with circadian rhythms of inflammatory markers in patients with RA [127]. This suggests that diet–microbiome interactions in RA may depend not only on nutrient quality but also on temporal patterns of food intake, although this remains an emerging field requiring further validation.

Overall, direct evidence linking dietary intervention, microbiome modulation, and clinical improvement in RA remains limited. Future studies should combine dietary assessment with longitudinal microbiome profiling, metabolite measurements, and immune clinical endpoints to clarify whether microbiome changes mediate the effects of dietary strategies in RA.

## 5. Conclusions

Dietary and lifestyle changes can be valuable adjuncts to conventional therapy and may also contribute to RA prevention. Smoking, physical activity, diet, and gut microbiota are listed among factors with the greatest impact on severity and risk of developing RA. Among these modifiable factors, diet and gut microbiome appear particularly important because they may influence not only metabolic health but also immune–inflammatory pathways relevant to RA pathogenesis and progression. At the same time, the current evidence remains heterogeneous, and in many areas, it is still insufficient to support precise clinical recommendations.

The current evidence suggests that specific compounds and supplements—particularly omega-3 fatty acids and vitamin D, as well as whole dietary regimens, such as Mediterranean diet or vegan diet, may be beneficial in managing RA symptoms. In several studies, omega-3 supplementation resulted in decreased intake of NSAID or improvement in some symptoms such as morning stiffness and joint tenderness. It has also been shown that vitamin D levels might be inversely associated with RA activity parameters, such as DAS28. Following the Mediterranean diet or vegan diet might lead to reduction in inflammation and alleviate some symptoms of the disease. However, for most dietary interventions, the overall quality of evidence remains low-to-moderate, as many studies are small, short-term, methodologically heterogeneous, and sometimes limited to selected populations such as women only or patients undergoing complex multicomponent interventions. This makes it difficult to distinguish diet-specific effects from broader lifestyle-related changes and limits the applicability of the observed findings.

Available studies suggest that the gut microbiota of RA patients changes in the course of disease and in response to treatment. RA patients display diminished gut microbial diversity, which might correlate with disease duration and autoantibody levels. Beyond taxonomic alterations, emerging evidence highlights the importance of functional features of the gut microbiome, including microbial metabolites, intestinal barrier integrity, and translocation of bacterial components, which may contribute to immune activation in RA. According to the current evidence, there is an inseparable connection between diet composition and gut microbiota. Therefore, the modification of gut microbiome through dietary interventions or probiotic supplementation may become an essential part of RA therapy. Beneficial impact of the Mediterranean diet or plant-based diet on gut microbiome has already been demonstrated. Some studies also exhibited anti-inflammatory properties of probiotic supplementation in RA, particularly in terms of probiotics comprising *Lactobacillus* strains. Prebiotics and synbiotics have also shown modest but promising effects on disease activity and inflammatory markers in RA, although mechanistic data linking these interventions to specific microbiome changes remain limited. Furthermore, recent findings suggest that temporal aspects of diet, such as meal timing, may modulate gut microbial rhythmicity and inflammatory responses, adding another layer of complexity to diet–microbiome interactions in RA. Nevertheless, causal relationships between dietary modulation, microbiome changes, and clinical improvement in RA remain insufficiently established. In particular, only a minority of studies have simultaneously assessed dietary exposure or intervention, microbiome composition, microbial metabolites, immune markers, and clinical outcomes within the same design.

Future research should therefore move beyond descriptive associations and prioritize adequately powered, well-controlled longitudinal and interventional studies that integrate dietary assessment or dietary intervention with microbiome profiling, metabolomics, immune phenotyping, and standardized clinical endpoints. Such studies should aim to determine whether microbiome alterations mediate the clinical effects of diet rather than merely accompany them. Special attention should be given to functionally relevant microbial signatures, including taxa associated with inflammatory burden or treatment response, such as *Collinsella*, *Subdoligranulum*, and *Fusicatenibacter*, as well as to microbial pathways related to short-chain fatty acids, tryptophan metabolism, bile acid signaling, and intestinal barrier integrity. Rather than focusing only on taxonomic composition, future studies should identify reproducible microbial and metabolic profiles that could serve as biomarkers of response to dietary or microbiome-modulating interventions.

In the longer term, the incorporation of microbiome detection into individualized RA management may help identify patient subgroups more likely to benefit from specific nutritional strategies, probiotics/prebiotics, or combined microbiome-modulating approaches. However, such personalized application will require standardized sampling and analytical methods, validation in independent cohorts, and better understanding of strain-level and functional differences within the RA-associated microbiome. Thus, mutual dependencies of diet, microbiome, and RA activity or risk should be subjected to further comprehensive research.

## Figures and Tables

**Table 1:** Impact of vitamin D concentration on RA activity.

Study Design	Participants	Evaluated Parameters of Disease Activity	Main Results	Reference
Meta-analysis	2885 RA patients, 1084 controls	DAS28	Inverse association between vitamin D levels and RA activity in most included studies.	[7]
Meta-analysis	1143 RA patients, 963 controls	DAS28	Lower vitamin D levels in RA; negative correlation with DAS28.	[8]
Meta-analysis	3489 RA patients	DAS28, CRP	Negative correlation between vitamin D and DAS28/CRP levels.	[9]
Cross-sectional	266 recent onset RA patients	DAS28	No significant association.	[10]
Cross-sectional	499 active RA patients	DAS28-ESR, DAS28-CRP, ESR, CRP, HAQ Score, patient assessment on VAS, evaluator assessment on VAS, pain on VAS, van der Heidje erosion score, vdHS score, joint space narrowing, CCP	No significant association with DAS28 or inflammatory markers.	[11]
Cross-sectional	181 RA patients, 186 controls	DAS28-ESR	No significant association.	[12]
Cross-sectional	35 RA patients, 38 controls	DAS28-ESR, joint damage (Steinbrocker criteria), serum IL-6	No significant association between 25(OH)D and DAS28. Positive correlation between 25(OH)D and IL-6.	[13]

CCP, cyclic citrullinated peptide; CRP, C-reactive protein; DAS28, 28-joint count disease activity score; ESR, erythrocyte sedimentation rate; HAQ, Health Assessment Questionnaire; IL-6, interleukin 6; VAS, visual analog scale; vdHS, van der Heijde–Sharp.

## Data Availability

Data sharing is not applicable.

## Abbreviations

The following abbreviations are used in this manuscript:

AA	arachidonic acid
ACPA	anti-citrullinated protein autoantibody
ACR	American College of Rheumatology response criteria
AID	anti-inflammatory diet
CAT	catalase
CCP	cyclic citrullinated peptide
CDAI	Clinical Disease Activity Index
CRP	C-reactive protein
DAS28	28-joint count disease activity score
DHA	docosahexaenoic acid
DMARD	disease-modifying anti-rheumatic drug
EMS	early morning stiffness
EPA	eicosapentaenoic acid
ESR	erythrocyte sedimentation rate
GPx	glutathione peroxidase
HAQ	Health Assessment Questionnaire
HAQ-DI	Health Assessment Questionnaire Disability Index
hs-CRP	high-sensitivity C-reactive protein
IL-1	interleukin 1
IL-1β	interleukin 1β
IL-6	interleukin 6
IL-9	interleukin 9
IL-12	interleukin 12
IL-17	interleukin 17
IL-23	interleukin 23
mHAQ	modified Health Assessment Questionnaire
NSAID	nonsteroidal anti-inflammatory drug
PCBs	polychlorinated biphenyls
RA	rheumatoid arthritis
RAI	Ritchie’s articular index
RAI^1^	relative activity index
RF	rheumatoid factor
SCFA	short-chain fatty acid
SF-36	short form-36 health survey
SOD	superoxide dismutase
TGFβ1	transforming growth factor β1
Th17	T helper 17
t.i.d.	three times a day
TNF-α	tumor necrosis factor α
Treg	regulatory T cells
VAS	visual analog scale
vdHS	van der Heijde–Sharp
WBC	white blood cell
